# Case Report: Ovarian pregnancy, a rare but lethal condition: An analysis of 112 cases

**DOI:** 10.3389/fsurg.2023.1062228

**Published:** 2023-02-17

**Authors:** Mengyuan Shao, Xinyi Wang, Xin Zhou

**Affiliations:** Department of Obstetrics and Gynecology, Shengjing Hospital of China Medical University, Shenyang, China

**Keywords:** ovarian ectopic pregnancy, ultrasonic classification, laparoscopy surgery, reproductive outcomes, risk factors

## Abstract

**Study Objective:**

To explore how to improve the treatment, and prognosis of ovarian pregnancy (OP).

**Patients:**

A total of 111 OP patients, one of the patients suffered from OP twice.

**Results:**

In this study, 112 OP cases confirmed by postoperative pathology were retrospectively analyzed. Common risk factors for OP were previous abdominal surgery (39.29%) and intrauterine device use (18.75%). We modified the ultrasonic classification into four types: gestational sac type, hematoma type I, hematoma type II, and intraperitoneal hemorrhage type. Among these four types, the proportion of patients who underwent emergency surgery as initial treatment after admission was 68.75%, 10.00%, 92.00%, and 81.36%, respectively. The treatment for hematoma type I patients was often delayed. The rate of OP rupture was 86.61%. All methotrexate therapy for OP patients failed. All these 112 cases underwent surgery treatment finally. The surgical procedures were pregnancy ectomy and ovarian reconstruction by laparoscopy or laparotomy. No significant differences were observed in the operation time or intraoperative blood loss between laparoscopy and laparotomy. Laparoscopy showed less influence on patients regarding length of hospital stay and postoperative fever than laparotomy. Further, 49 patients who desired fertility were followed up over 3 years. Among them, 24 (48.98%) experienced spontaneous intrauterine pregnancy.

**Conclusion:**

Among the four modified ultrasonic classifications, hematoma type I was associated with more delays in surgical time. Laparoscopic surgery was a better choice for OP treatment. The reproductive prognosis of OP patients was promising.

## Introduction

Ovarian pregnancy (OP) is a rare and fatal type of ectopic pregnancy, accounting for approximately 0.5%–3% of all ectopic pregnancies (1, 2). The risk factors of OP include intrauterine device, assisted reproductive technology, pelvic inflammatory disease, etc. (3, 4). The clinical manifestations of OP, including amenorrhea, abdominal pain and vaginal bleeding, have no obvious specificity compared with tubal pregnancy (TP) (5). Due to the high vascularity of ovarian tissue (6), OP patients commonly develop intraperitoneal bleeding, which can progress to life-threatening hemorrhagic shock resulting from pregnancy sac rupture. A previous study showed that the rupture rate of OP patients was 56.34% (7), which was significantly higher than that of TP (8).

As both OP and TP generally present as adnexal masses, differential diagnosis between OP and TP is challenging. Three-dimensional pelvic ultrasound and serum beta subunit of human chorionic gonadotropin (*β*-hCG) are widely used auxiliary tests. Detecting the location of unruptured OP masses by ultrasound is possible; however, detecting the location of ruptured masses of OP is difficult. A previous study reported that OP patients had higher serum *β*-hCG levels than TP patients (7), however, due to rareness of the disease and limited data, this conclusion need to be further validated by research with more cases.

Surgery is the main treatment for OP. With the development of technology, laparoscopy has gradually become the standard method for diagnosis and treatment of OP (9). Compared with laparotomy, laparoscopic surgery has advantages of shorter hospital stay, faster recovery of the patient and less postoperative adhesion. Although the original surgery for OP was ipsilateral oophorectomy, conservative ovarian surgery, such as cystectomy or wedge resection has become the recommended management for OP patients to preserve fertility (10). In addition, methotrexate (MTX) treatment for OP patients remains controversial (11).

Herein, we conducted a retrospective analysis of 112 OP cases, including analyzing the clinical characteristics, the management and reproductive outcomes, etc. We also explored how to avoid mistreatment of OP, which may be a life-threatening condition.

## Materials and methods

A retrospective study of 111 OP patients was conducted in Shengjing Hospital of China Medical University. Notably, one of the patients suffered from OP twice within 2 years. Therefore, 112 total cases were included in this study. All cases fulfilled the Spiegelberg criteria: (1) the gestational sac is located in the region of the ovary; (2) the ectopic pregnancy is attached to the uterus by the ovarian ligament; (3) ovarian tissue in the wall of the gestational sac is proved histologically; and (4) the tube on the involved side is intact (12).

The following information of each case was collected: age, clinical manifestations, pregnancy history, previous surgical history, physical signs, ultrasound findings, serum *β*-hCG levels, management strategies and outcomes. A previous study classified 12 OP cases into ruptured and unruptured type based on ultrasound findings (13). We modified this classification into four categories: (1) gestational sac type: a typical gestational sac in ovary; (2) hematoma type I: adnexal mass < 3.5 cm; (3) hematoma type II: adnexal mass ≥ 3.5 cm; and (4) intraperitoneal hemorrhage type: hemorrhagic effusion in the pelvic or abdominal cavities. Among all these 112 cases, some patients underwent surgery as initial treatment (within 24 h after admission) and other patients underwent surgery after failure of conservative therapy, including expectant therapy and MTX therapy. Eighty-nine patients were followed up, and their postoperative pregnancy experience were reviewed.

For statistical analysis, Student's t-test and a chi-square test were performed using the Statistical Package for Social Science (SPSS) version 26 (IBM Corp, United States).

This study was approved by the Ethics Committee of Shengjing Hospital of China Medical University under the approval number 2022PS739k.

## Results

### Basic information

The mean age of the 112 cases was 30 ± 5 years. The mean gravidity and parity of the cases were 2.13 ± 1.38 and 0.63 ± 0.61. Further, eight cases had no prior history of pregnancy, whereas two had a history of ectopic pregnancy. Among 64 cases who had delivery history, 29 delivered vaginally and 35 underwent a cesarean section. Among the 112 cases, 103 conceived naturally and nine conceived using assisted reproductive technology (ART) in this ovarian pregnancy.

### Risk factors

The proportions of risk factors, such as history of abdominal surgery, intrauterine devices use, and ART use were 39.29%, 18.75%, and 8.04%, respectively. Regarding other potential risk factors for OP, four (3.57%), two (1.79%), and two (1.79%) cases had endometriosis, a history of previous ectopic pregnancy, and uterine malformation, respectively.

### Clinical presentations

Classic clinical symptoms of OP included abdominal pain (100 cases, 89.29%), amenorrhea (92 cases, 82.14%), and vaginal bleeding (46 cases, 41.07%). Further, 17 cases (15.18%) developed hemorrhagic shock.

**Figure 1:**
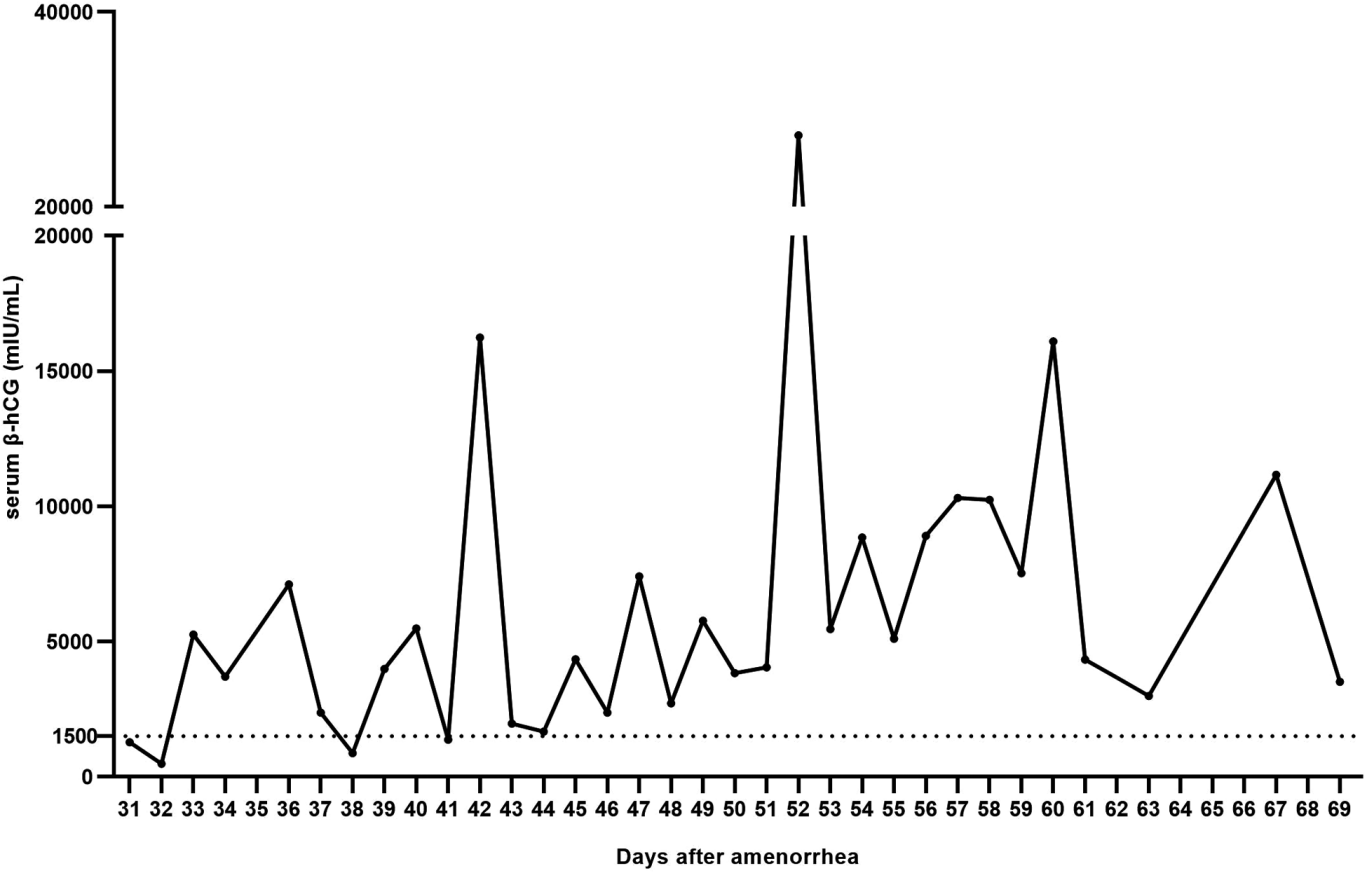
Mean serum *β*-hCG levels of ovarian pregnancy patients on different days after amenorrhea. Two ovarian pregnancy cases with intrauterine pregnancy were excluded. Most of the mean values exceeded 1500 mIU/ml, and the highest and the lowest mean levels were 27,353 mIU/ml (52 days after amenorrhea) and 478.025 mIU/ml (32 days after amenorrhea), respectively.

Serum *β*-hCG levels of OP cases (excluding two OP cases with intrauterine pregnancy) ranged from 60.08 mIU/ml to 43,324 mIU/ml (mean, 6464.42 ± 8409.31 mIU/ml). Mean serum *β*-hCG levels were calculated according to the days after amenorrhea and most of the mean *β*-hCG levels exceeded 1500 mIU/ml. The highest and lowest mean levels of serum *β*-hCG were 27,353 mIU/ml (52 days after amenorrhea) and 478.025 mIU/ml (32 days after amenorrhea), respectively. Mean serum *β*-hCG levels were 3492.49 ± 3103.83, 6766.98 ± 9393.77, and 9561.26 ± 10,351.91 mIU/ml during 30–40, 41–50, and 51–69 days of amenorrhea, respectively (Figure 1).

**Figure 2:**
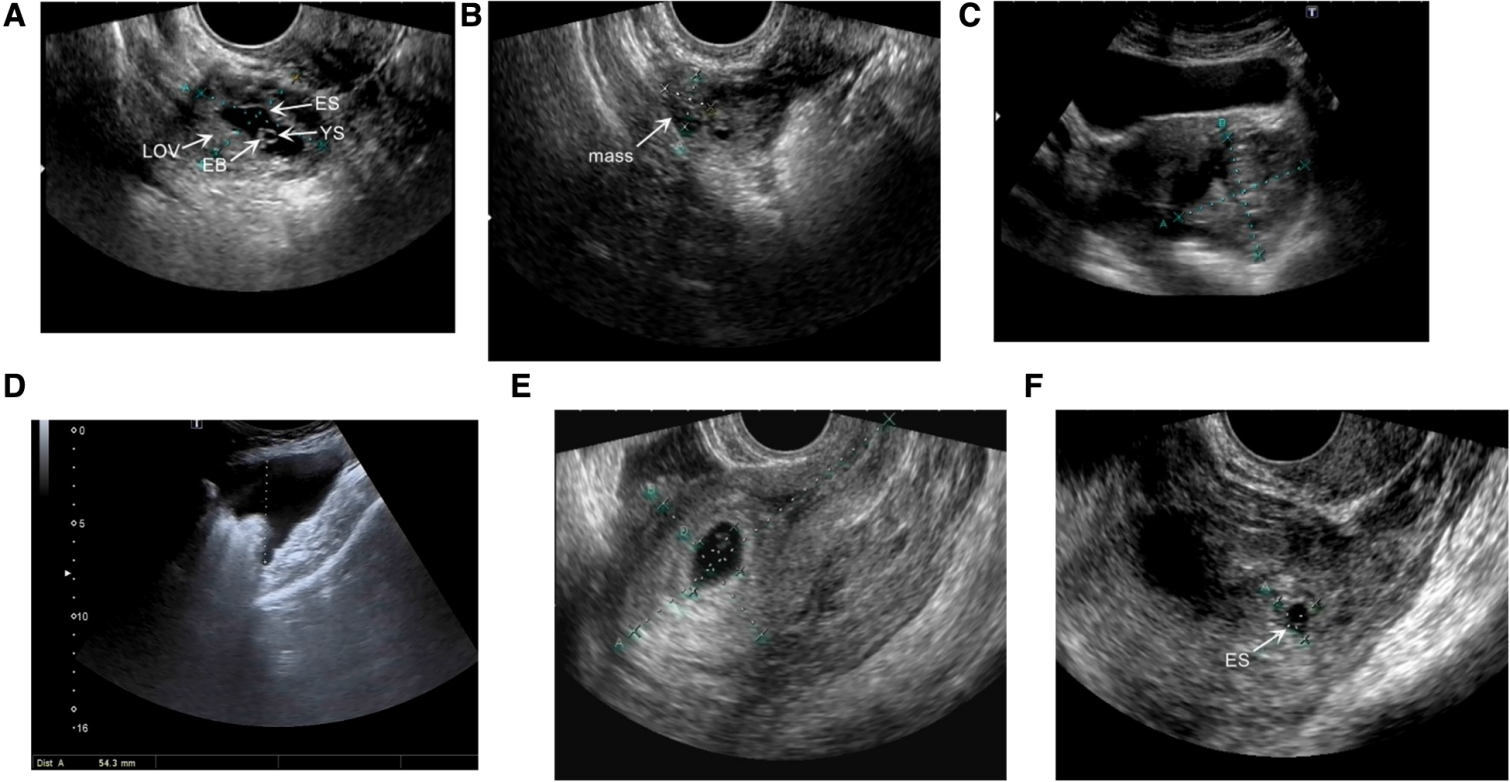
Ultrasound images showing several types of ovarian pregnancy. (**A**) Gestational sac type: ovarian tissue (LOV) can be seen surrounding the embryo sac (ES). The yolk sac (YS) and embryo bud (EB) are located in the ES. (**B**) Hematoma type I: a mixed echoic mass approximately 2.1 × 2.3 × 1.9 cm in size. (**C**) Hematoma type II: a mixed echoic mass approximately 6.0 × 5.3 cm in size. (**D**) Intraperitoneal hemorrhage type: 5.4 cm deep pelvic effusion. (**E, F**) An ovarian pregnancy patient complicated with intrauterine pregnancy. (**E**) The intrauterine embryo sac. (**F**) ES in the adnexal mass.

We modified ultrasonic classification into four types and typical images are shown in Figure 2. Intraperitoneal hemorrhage was the most common type, comprising 59 cases (52.7%). Among the four types, the mean serum *β*-hCG level of the gestational sac type, 12,630.31 ± 10,272.06 mIU/ml, was the highest (Table 1). Of 10 hematoma type I cases, eight had serum *β*-hCG level > 1500 mIU/ml and two had serum *β*-hCG level < 1500 mIU/ml.

**Table 1:** Cases involving different treatment methods, serum *β*-hCG levels and characteristics of previous surgeries of four categories of ultrasonographic manifestations.

Categories	Number^a^	Surgery for initial treatment^b^	Conservative management for initial treatment	Mean value of serum *β*-hCG (mIU/ml)	Number of patients who underwent previous surgery
Gestational sac type	16	11 (68.75%)	5 (31.25%)	12,630.31 ± 10,272.06	4 (25.00%)
Hematoma type I	10	1 (10.00%)	9 (90.00%)	3401.45 ± 2765.46	2 (20.00%)
Hematoma type II	25	23 (92.00%)	2 (8.00%)	7134.87 ± 9108.21	14 (56.00%)
Intraperitoneal hemorrhage type	59	48 (81.36%)	11 (18.64%)	4267.87 ± 5292.59	27 (45.76%)

^a^ 110 cases with ovarian pregnancy were included after two ovarian pregnancy cases complicated with intrauterine pregnancy were excluded.

^b^ Indicates that surgery was performed within 24 h after admission.

### Treatment strategies

Of the 112 OP cases, 85 underwent surgery as initial treatment after admission. Three cases underwent surgery after failure of MTX and 24 cases underwent surgery after failure of expectant therapy. The three cases underwent MTX therapy were monitored in the hospital; however, the therapy failed in all three cases. Among these three cases, two were observed 7 days after intramuscular MTX injection, and the serum *β*-hCG level decrease was not observed as expected so that surgery was performed. Signs of mass rupture were observed in the remaining one case on the fifth day after the second MTX injection; therefore, emergency surgery was performed.

From the perspective of ultrasonic classification, there were 10 cases with hematoma type I. Of these ten cases, one patient underwent surgery as initial treatment, and the other nine cases underwent conservative therapy as initial treatment. Among 25 cases with hematoma type II, 23 cases underwent surgery as initial treatment.

**Figure 3:**
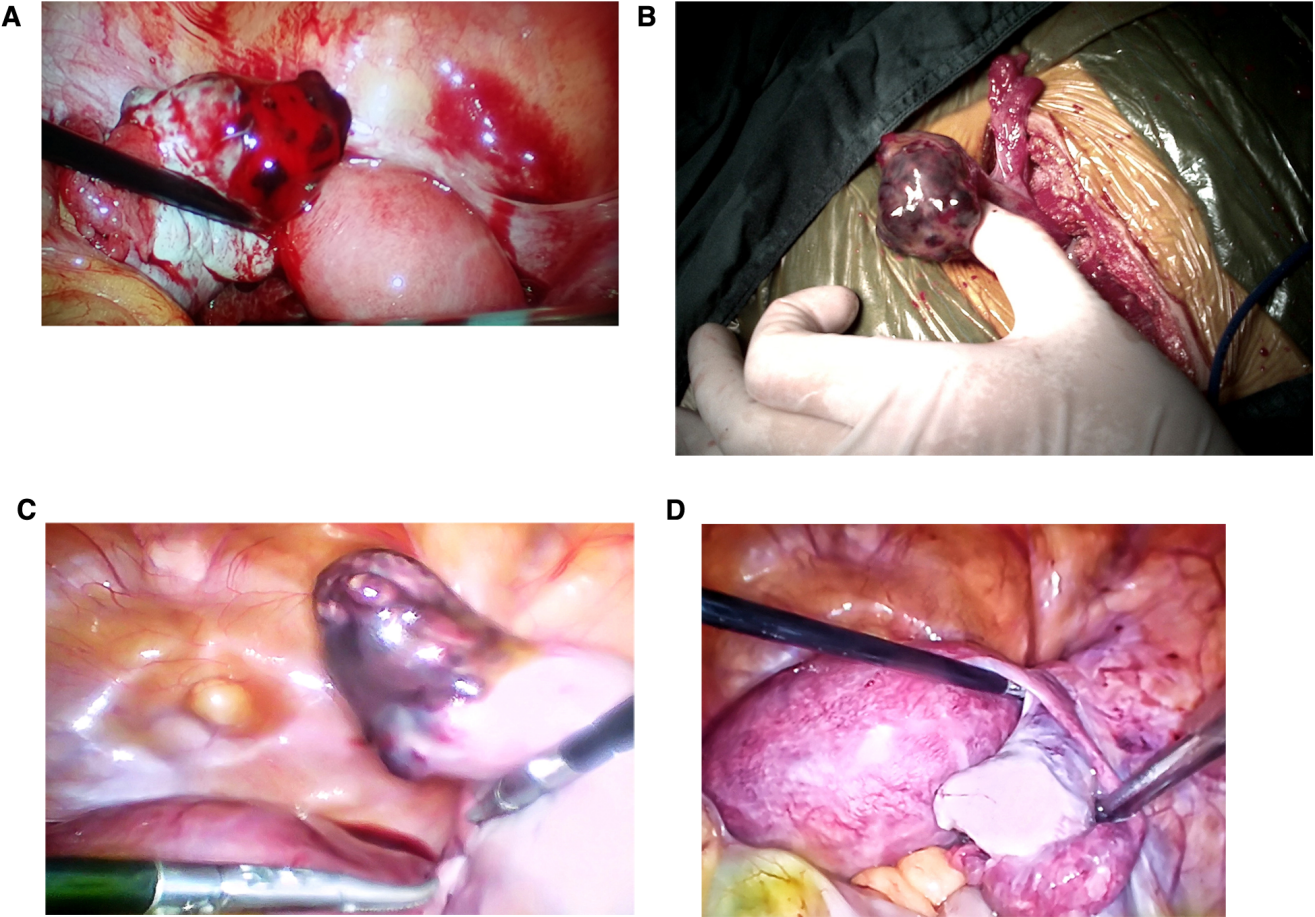
Intraoperative photographs of ovarian pregnancy patients: (**A**) laparoscopic image of a ruptured ovarian pregnancy lesion. (**B**) Image of an unruptured ovarian pregnancy through laparotomy. (**C**) and (**D**) show the graph in the same patient. (**C**) Laparoscopic image of an ovarian pregnancy lesion. (**D**) The reconstructed ovary tissue after pregnancy ectomy.

The surgical procedures were pregnancy ectomy and ovarian reconstruction in all of the cases. Intraoperative findings are shown in Figure 3. According to surgical exploration findings, it was found that the incidence of OP rupture was 86.61%. Seventy cases (62.5%) underwent laparoscopic surgery and 42 (37.5%) underwent laparotomy. There were no significant differences in operation time (87.17 ± 38.85 min vs. 80.95 ± 29.47 min, *p *> 0.05) or intraoperative blood loss (81.54 ± 210.52 ml vs. 75.00 ± 162.15 ml, *p *> 0.05) between laparoscopic surgery and laparotomy. Shorter hospital stay (5.07 ± 1.54 days vs. 5.60 ± 0.86 days, *p *= 0.04) and lower rate of post-operation fever (temperature over 37.4 °C within 72 h after surgery) (21.4% vs. 59.5%, *p* < 0.001) were found in patients who underwent laparoscopic surgery. From January 2011 to January 2016, 54.10% cases (33/61) underwent laparoscopic surgery, whereas 72.55% cases (37/51) underwent laparoscopic surgery from January 2016 to January 2021.

### Special cases

Persistent ectopic pregnancy after pregnancy ectomy and ovarian reconstruction occurred in one OP patient with a preoperative serum *β*-hCG level of 5578 mIU/ml. The serum *β*-hCG level decreased gradually following surgery to 533.54 mIU/ml on the 12th day post-surgery. However, the *β*-hCG level elevated to 711.04 mIU/ml on the 17th day post-surgery. Ultrasound examination revealed a 3.8 × 1.6 × 1.5 cm right adnexal heterogeneous mass. MTX treatment was administered, and the *β*-hCG level decreased to 54.80 mIU/m on the 21st day.

Two OP cases with intrauterine pregnancies maintained their pregnancies to full-term delivery successfully after surgery (Figures 2E,F).

Further, one patient suffered from ovary pregnancies twice in 2 years, which occurred in both sides of ovaries. Therefore, laparoscopic pregnancy ectomy and ovarian reconstruction were performed twice. Additionally, one year after the second surgery, the patient successfully conceived *via* ART and delivered two children.

### Reproductive outcomes

The reproductive prognosis of 89 patients was followed up and 22 patients were lost. Among these 89 patients, 56 patients had desire of fertility. Forty-nine of 56 patients were followed up over 3 years, while in these 49 patients, 24 (48.98%) conceived spontaneous intrauterine pregnancies, five conceived intrauterine pregnancies *via* ART, two had ectopic pregnancies, and 18 failed to conceive. Subsequent spontaneous intrauterine pregnancy was observed in 13 of the 29 patients who underwent laparoscopic surgery, compared with 11 of the 20 patients who underwent laparotomy. There were no significant differences in reproductive outcomes between laparoscopic surgery and laparotomy (*p *> 0.05).

## Discussion

We discovered that the percentage of OP rupture was 86.61%, which is significantly higher than that of TP rupture (reported previously as 15.97%) (14). Misdiagnosing OP as TP may lead to conservative treatment selection, gestational sac rupture and intraperitoneal bleeding, which are life-threatening. Although MTX therapy was effective in some TP patients, its safety and efficacy remain unclear in OP patients (11, 15–17). Therefore, timeous surgery is the key to OP treatment. Improving our clinical understanding of OP is helpful to reduce the risk of delayed treatment.

In this study, the mean *β*-hCG level at the time of admission was 6464.42 mIU/ml in OP patients, which was significantly higher than that of TP patients reported previously (1034.20 mIU/ml) (18). In addition, the mean *β*-hCG value of most OP patients exceeded 1500 mIU/ml. Thus, a higher *β*-hCG level in ectopic pregnancy may indicate OP in clinical settings. This may be due to a rich blood supply to the ovary; therefore, a pregnancy sac implanting in the ovary receives more nutrition and develops better than in fallopian tube.

In this study, we modified ultrasound classification of 112 OP cases into four types. It was observed that most cases with the gestational sac type, hematoma type II, and intraperitoneal hemorrhage type received timeous surgical treatment, whereas only 10.0% of patients of hematoma type I underwent surgery as the initial treatment. Therefore, OP cases of hematoma type I are more likely to experience treatment delay. Although serum *β*-hCG levels in 70.0% of hematoma type I patients exceeded 1,500 mIU/ml (range, 3282–26,244 mIU/ml), which did not meet the indication for expectant treatment according to 2019 NICE guideline (19), all these patients received expectant treatment as initial therapy. All hematoma type I patients who received expectant treatment underwent surgery due to mass enlargement or a continuous increase in serum *β*-hCG levels. As a result, for EP patients with an adnexal mass who do not meet the indications for conservative treatment, the indications must be followed strictly to avoid delayed treatment.

Since the reports on medically managed OP is limited, the safety and efficacy of MTX treatment for OP remains unclear. In this study, three OP patients received MTX therapy as initial treatment but all failed. Due to the limited clinical cases at present, the data of these 3 cases are also of reference value. However, one patient with persistent ectopic pregnancy was successfully treated by MTX treatment, which means MTX therapy may be effective in treating persistent ectopic pregnancy after surgery in OP patients. Additionally, 24 patients who chose expectant treatment as their first choice underwent surgery finally. Therefore, first-choice treatment for OP patients may be timeous surgery rather than conservative treatment.

The use of laparoscopic surgery for OP patients is gradually increasing. In current study, the proportion of laparoscopic surgery was 72.55% in the later 5 years, compared with 54.10% in the early 5 years. Bipolar coagulation during laparoscopic surgery may cause impaired ovarian function due to thermal radiation, which raises concern regarding ovarian and reproductive function in OP patients (20). In our study, there was no significant difference in reproductive outcomes between patients who underwent laparotomy and those who underwent laparoscopic surgery. Given the advantages of minimally invasive laparoscopic surgery, including prompt postoperative recovery and short hospital stay, laparoscopic surgery is a better choice for OP patients.

Studies on the postoperative reproductive outcomes of OP patients are limited. The proportion of spontaneous intrauterine conception in OP patients in this study was 48.98%, which is similar with 37.03%–65.38% reported previously (1, 21, 22). Therefore, based on our data, the reproductive prognosis of OP patients is promising but requires further exploration.

## Conclusion

Commonly misdiagnosed as TP, OP is a rare ectopic pregnancy with a high risk of intraperitoneal bleeding. Mean serum *β*-hCG levels of OP patients were calculated according to the days after amenorrhea and most of them exceeded 1,500 mIU/ml. Hematoma type I patients experience treatment delays more commonly than other three types due to a lack of strict adherence to indications for conservative treatment. Laparoscopic surgery is a better choice than laparotomy for OP treatment. The reproductive prognosis of OP patients is promising.

## Data Availability

The original contributions presented in the study are included in the article/Supplementary Material, further inquiries can be directed to the corresponding author.
